# Susceptibility‐Guided Versus Empirical Therapy for *Helicobacter pylori* Infection in a High Resistance Setting: A Randomised Controlled Trial

**DOI:** 10.1002/mbo3.70313

**Published:** 2026-05-17

**Authors:** Kemei Lu, Xuefei Zou, Cuicui Lang, Lina Zang, Xuechao Yang, Weiwei Sang, Jinyan Wang, Qian Feng, Ying Mu, Lifeng Liu, Chunhong Xu, Jingrun Zhao

**Affiliations:** ^1^ Department of Gastroenterology Liaocheng People's Hospital Liaocheng Shandong Province China; ^2^ Department of Gastroenterology Shandong Province Maternal and Child Health Care Hospital Jinan Shandong Province China; ^3^ Department of Gastroenterology Central Hospital affiliated to Shandong First Medical University Jinan Shandong Province China

**Keywords:** antibiotic resistance, antimicrobial susceptibility testing, *Helicobacter pylori*, polymerase chain reaction

## Abstract

Clarithromycin resistance is a major determinant of treatment failure, and the benefits of susceptibility‐guided therapy in high‐resistance settings remains incompletely understood. To evaluate the clinical effectiveness of the susceptibility‐guided compared with the empirical therapy in treatment‐naïve *H. pylori* infection in a high resistance setting. In this single‐centre, randomised, superiority trial, 500 treatment‐naïve adults were allocated (1:1) to susceptibility‐guided sequential therapy (SGT) or empirical clarithromycin ‐containing quadruple therapy (ET). Clarithromycin and levofloxacin antimicrobial resistance were determined using PCR. The primary endpoint was first‐line eradication in the intention‐to‐treat (ITT) population. Sensitivity analyses were performed using complete‐case and multiple imputation approaches. Among the 494 analysed participants (244 SGT, 250 ET), resistance to clarithromycin and levofloxacin was 69.3% and 51.6%, respectively. In the ITT analysis, first‐line eradication was achieved in 59.8% of participants in the SGT group and 62.8% in the ET group [absolute risk difference −3.0%; odds ratio (OR) = 0.882, 95% confidence interval (CI, 0.614–1.268), *p* = 0.499]. Per‐protocol eradication rates were 84.4% and 89.7%, respectively [absolute risk difference −5.3%; OR = 0.620, 95% *CI* (0.328–1.173), *p* = 0.139]. Notably, despite a clarithromycin resistance rate approaching 70%, empirical clarithromycin‐containing quadruple therapy achieved a high per‐protocol eradication rate. Sensitivity analyses yielded consistent results. In a high resistance region, PCR‐based susceptibility‐guided sequential therapy did not demonstrate superiority over empirical treatment. However, empirical clarithromycin ‐containing quadruple therapy maintained satisfactory efficacy. Overall, these findings have implications for treatment strategy selection in high‐resistance microbiological settings.

**Trial Registration:** ClinicalTrials. gov Identifier: NCT05549115

## Graphical abstract

In a randomised trial conducted in a high‐resistance region, PCR‐based susceptibility‐guided therapy showed no superiority over empirical therapy for *Helicobacter pylori* eradication. Empirical clarithromycin‐containing quadruple therapy remained effective despite high clarithromycin resistance.

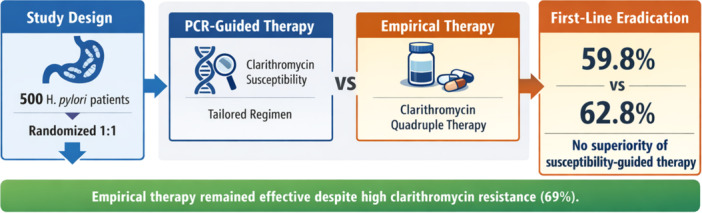

## 1 Introduction

*Helicobacter pylori* infects more than half of the global population and consistently induces chronic gastritis, which may progress to severe complications such as peptic ulcer disease, gastric adenocarcinoma, and gastric mucosa‐associated lymphoid tissue (MALT) lymphoma (Malfertheiner et al. 2022). Eradication therapy effectively controls disease progression and reduces the risk of these outcomes (Zhou et al. 2022). However, the increasing prevalence of antimicrobial resistance has become a major barrier to successful eradication.

International guidelines differ regarding the timing of antimicrobial susceptibility testing (AST) implementation (Malfertheiner et al. 2022; Zhou et al. 2022; Smith et al. 2024; Chey et al. 2024). AST after treatment failure may have limited clinical impact, as clarithromycin resistance is common following exposure and alternative regimens are typically selected empirically. Resistance to amoxicillin and tetracycline remains consistently low and stable in the Asia‐Pacific region (6% and 4% respectively), (Hong et al. 2024); therefore, routine AST for these drugs is unnecessary (Salahi‐Niri et al. 2024). Resistance to metronidazole is common; however, it does not influence the choice of bismuth quadruple therapy as it is unaffected by metronidazole resistance (Malfertheiner et al. 2022). In contrast, levofloxacin resistance is rapidly increasing worldwide and represents the most critical determinant of treatment efficacy, highlighting the importance of AST‐based regimen selection (Schulz et al. 2025).

Currently, two main AST approaches are available. The traditional gold standard is culture‐based testing of gastric biopsy samples for susceptibility to amoxicillin, clarithromycin, metronidazole, tetracycline, and levofloxacin. However, isolating *H. pylori* from biopsies is technically challenging and not always successful, which limits its practical use (Rajadurai and Moss 2025). In contrast, molecular methods—particularly PCR‐based assays—have been increasingly adopted to detect clarithromycin and levofloxacin resistance, which are both highly prevalent and clinically relevant. Compared with culture‐based methods, PCR is more reproducible, rapid, and cost‐efficient, making it better suited for routine clinical practice (Francesco et al. 2022).

In China, clarithromycin‐ and levofloxacin‐containing regimens are the most commonly used empirical therapies in first‐ and second‐line settings, respectively (Song et al. 2022). The rates of resistance to clarithromycin and levofloxacin are 30% and 36%, respectively, (Hong et al. 2024); therefore, tailoring treatment according to AST is particularly important. In this study, we conducted a prospective, randomised controlled trial to compare a susceptibility‐guided sequential strategy—using PCR‐based AST to guide both first‐line and rescue therapies—with standard empirical treatment. Specifically, clarithromycin susceptibility was used to guide first‐line treatment, while levofloxacin susceptibility informed rescue regimens. The regimens were selected considering local susceptibility patterns, updated guidelines, and recent randomised controlled trials (Zhou et al. 2022; Yin et al. 2022; Li et al. 2021). We hypothesise that this susceptibility‐guided sequential strategy would achieve a higher eradication rate compared with empirical therapy.

## 2 Participants and Methods

### 2.1 Study Design and Ethics

This was a prospective, single‐centre, randomised, open‐label, active‐controlled superiority trial with a 1:1 allocation ratio (ClinicalTrials.gov identifier: NCT05549115). A detailed description of the study design has been described in a previous article (Lu et al. 2023). The study was conducted in accordance with the Declaration of Helsinki. Ethical approval was obtained from the Medical Ethics Committee of Liaocheng People's Hospital (approval number: 2022190). Written informed consent was obtained from all participants prior to enrolment.

### 2.2 Study Participants

Eligible participants were men and women aged 18–75 years with confirmed *H. pylori* infection, diagnosed by at least one of the following tests within 4 weeks: 13 C/14C‐urea breath test (UBT), stool antigen test, rapid urease test, or histological analysis. Participants were treatment‐naïve for *H. pylori*.

Exclusion criteria included: (1) allergy to any study drugs; (2) pregnancy or lactation; (3) severe systemic disease (e.g., cardiopulmonary or hepatic dysfunction); and (4) active peptic ulcer within 8 weeks, history of gastric cancer, or prior gastrectomy.

### 2.3 Sample Size and Randomisation

Sample size was calculated for a superiority design. Based on meta‐analysis data (Ouyang et al. 2022; Huang et al. 2022), we assumed an eradication rate of 90% for susceptibility‐guided therapy and 80% for empirical therapy, with a two‐sided α of 0.05% and 80% power to detect a 10% absolute difference between groups. The required sample size was 197 participants per group. Allowing for a 20% dropout rate, we planned to recruit 500 participants (250 per arm).

Randomisation was performed using a computer‐generated random number table in IBM SPSS Statistics for Windows, version 22.0 (IBM Corp., Armonk, NY, USA). Participants were allocated in a 1:1 ratio to the susceptibility‐guided therapy (SGT) group or the empirical therapy (ET) group. This was an open‐label trial; therefore, blinding of participants and investigators was not feasible. However, outcome assessors and data analysts remained blinded to treatment allocation.

### 2.4 Antimicrobial Susceptibility Testing

Gastric mucosal biopsy specimens were collected, stored and transferred under a standardised procedure (Lu et al. 2023). Two biopsies were taken from each participant: one from the greater curvature of the antrum and one from the lesser curvature of the gastric body. Antimicrobial susceptibility testing was conducted by PCR (Zhiyuan Medical Inspection Institute Co. Ltd., Hangzhou, China), targeting mutations associated with clarithromycin and levofloxacin resistance. The detailed PCR protocol is provided in Additional File 1.

### 2.5 Interventions

#### 2.5.1 Empirical Therapy (ET) Group

Participants received clarithromycin quadruple therapy as first‐line treatment for 14 days: esomeprazole 20 mg twice daily, colloidal bismuth pectin 150 mg four times daily, clarithromycin 500 mg twice daily, and amoxicillin 1.0 g twice daily. The combination of amoxicillin and clarithromycin regimen (74.8%) is the most commonly prescribed treatment in China (Song et al. 2022). Participants who failed first‐line treatment were given high‐dose dual therapy (HDDT) as rescue treatment: esomeprazole 20 mg four times daily and amoxicillin 750 mg four times daily for 14 days (Bi et al. 2022; Shen et al. 2022).

#### 2.5.2 Susceptibility‐Guided Therapy (SGT) Group

Treatment regimens were tailored to *H. pylori* resistance patterns.

1. First‐Line Therapy
  ▪ Clarithromycin‐sensitive strains: clarithromycin triple therapy (esomeprazole 20 mg twice daily, clarithromycin 500 mg twice daily, and amoxicillin 1.0 g twice daily for 14 days).
  ▪ Clarithromycin‐resistant strains: HDDT as described above.

2. Rescue Therapy
  ▪ Levofloxacin‐sensitive strains: levofloxacin quadruple therapy (esomeprazole 20 mg twice daily, levofloxacin 500 mg once daily, amoxicillin 1.0 g twice daily, and colloidal bismuth pectin 150 mg four times daily for 14 days).
  ▪ Levofloxacin‐resistant strains: furazolidone quadruple therapy (esomeprazole 20 mg twice daily, furazolidone 100 mg twice daily, amoxicillin 1.0 g twice daily, and colloidal bismuth pectin 150 mg four times daily for 14 days) (Zhuge et al. 2018).

### 2.6 Outcomes

The primary outcome was the proportion of participants achieving successful *H. pylori* eradication, assessed by 13C‐UBT performed at least 4 weeks after first‐line therapy in both groups.

Secondary outcomes included:

1. Rescue therapy eradication rates and overall eradication rates in both groups.
2. Eradication rate of HDDT among participants with clarithromycin resistance and among ET participants who failed clarithromycin quadruple therapy.
3. Frequency of adverse events and treatment compliance in both groups.

### 2.7 Statistical Analysis

Continuous data are presented as a mean ± SD. Categorical data are presented as counts and proportions. Eradication rates were analysed using ITT (for all randomised participants), and per protocol (PP) (for participants with good compliance who completed 13C‐UBT follow‐up) analyses. Participants without follow‐up 13C‐UBT were considered treatment failures (‘not eradicated’). The eradication rates were compared between the two groups with the *χ*2 test or Fisher's exact probability test if the validity criteria of the *χ*2 test were not fulfilled, and the odds ratios (ORs) were calculated together with their 95% confidence intervals (CIs). In addition, A sensitivity analysis was performed using complete‐case analysis, a multiple imputation approach, and extreme‐case scenario analyses for missing data. Statistical significance was set at *p* < 0.05. All statistical analyses were performed using IBM SPSS Statistics for Windows, version 22.0 (IBM Corp., Armonk, NY, USA).

## 3 Results

### 3.1 Baseline Characteristics

**Figure 1:**
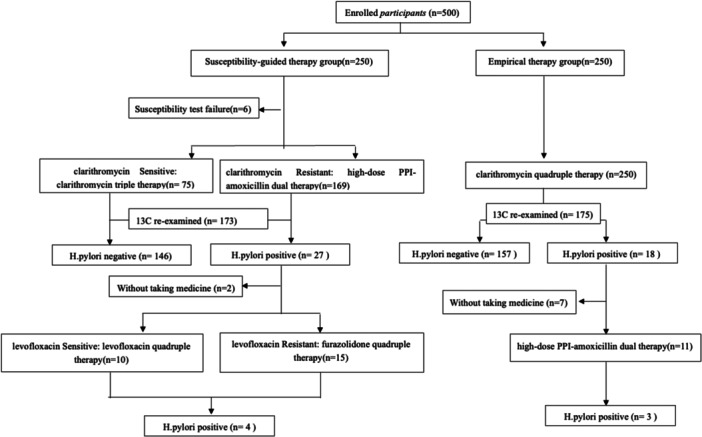
Study flowchart.

Between September 2022 and May 2024, a total of 500 treatment‐naïve *H. pylori*‐infected participants were enroled and randomised equally to the ET and SGT group (250 participants each). In the ET group, 116 participants were male and 134 were female, with a mean age of 45.2 ± 10.5 years. In the SGT group, 6 participants had no available AST results; thus, 244 participants were included in the analysis (95 males, 149 females; mean age 44.8 ± 10.4 years). There were no significant differences in baseline demographic characteristics between groups. The study flowchart is shown in Figure 1.

### 3.2 Antimicrobial Susceptibility Test Results

Among the 244 participants with AST results, clarithromycin resistance was observed in 169 (69.3%), levofloxacin resistance in 126 (51.6%), and dual resistance in 105 (43.0%). Detailed susceptibility profiles are shown in Table 1.

**Table 1:** Results of antimicrobial susceptibility testing (*n* = 244).

	Levofloxacin‐sensitive	Levofloxacin‐resistant	Total
Clarithromycin‐sensitive	54 (22.1%)	21 (8.6%)	75 (30.7%)
Clarithromycin‐resistant	64 (26.2%)	105 (43.0%)	169 (69.3%)
Total	118 (48.4%)	126 (51.6%)	244 (100%)

### 3.3 *H. pylori* Eradication Rates

In the ET Group, of the 250 participants, 1 discontinued due to elevated serum creatinine before starting therapy, 9 withdrew during treatment (2 drug allergy, 1 abdominal pain, 2 COVID‐19 infections, 4 received treatment for fewer than 10 days), 16 were lost to follow‐up, and 49 did not undergo post‐treatment 13C‐UBT. Among 175 participants re‐examined by 13C‐UBT, 157 achieved eradication and 18 remained positive. For 11 participants with rescue treatment, 8 achieved eradication and 3 remained positive.

In the SGT group, among 244 participants, 2 withdrew during treatment (1 drug allergy, 1 received treatment for fewer than 10 days), 15 were lost to follow‐up, and 54 did not undergo post‐treatment 13C‐UBT. Among 173 participants re‐examined by 13C‐UBT, 146 achieved eradication and 27 remained positive. For 25 participants with rescue treatment, 21 achieved eradication and 4 remained positive.

#### 3.3.1 Primary Endpoint

In the ITT analysis, first‐line treatment eradication rate was 59.8% (146/244) in the SGT group, as compared with 62.8% (157/250) in the ET group [OR = 0.882, 95% *CI* (0.614 ‐ 1.268), *p* = 0.499].

**Figure 2:**
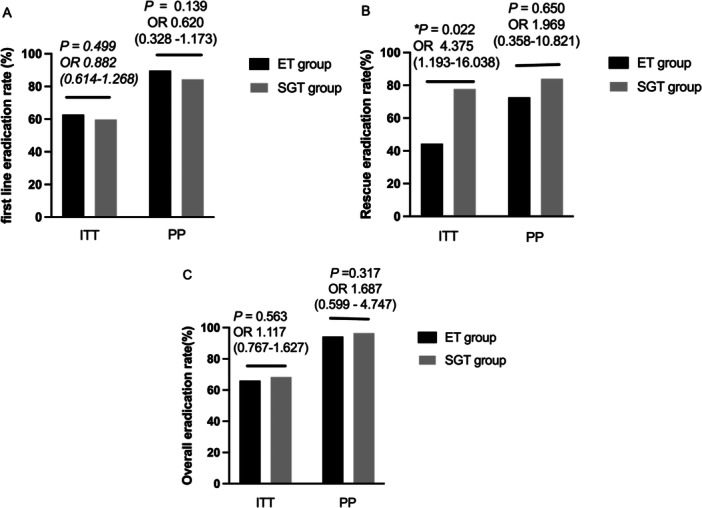
Eradication rates in the intention‐to‐treat and per‐protocol study population. (A) First line eradication rate; (B) Rescue treatment eradication rate; (C) Overall eradication rate.

According to the PP analysis, first‐line treatment eradication rate was 84.4% (146/173) in the SGT group, as compared with 89.7% (157/175) in the ET group with a OR of 0.620 [95% *CI* (0.328 ‐ 1.173), *p* = 0.139] (Figure 2A).

#### 3.3.2 Secondary Endpoints

In the ITT analysis, rescue treatment eradication rate was higher in the SGT group compared with the ET group [77.8% (21/27) vs. 44.4% (8/18)]. The difference was significantly [OR = 4.375, 95% CI (1.193–16.038), *p* = 0.022]. According to the PP analysis, rescue treatment eradication rate was higher in the SGT group compared with the ET group [84.0% (21/25) vs. 72.7% (8/11)], however, the difference was not significantly [OR = 1.969, 95% CI (0.358–10.821), *p* = 0.650] (Figure 2B).

In both the ITT and PP analyses, the overall eradication rate [ITT 68.4% (167/244) in the SGT group versus 66.0% (165/250) in the ET group, OR = 1.117, 95% *CI* (0.767‐1.627), *p* = 0.563]; PP 96.5% (167/173) in the SGT group versus 94.3% (165/175) in the ET group, OR = 1.687, 95% *CI* (0.599–4.747), *p* = 0.317] did not differ significantly between the two groups (Figure 2C).

### 3.4 Sensitivity Analyses

The extent and pattern of missing data were first evaluated. Among the study population, the overall proportion of missing data was 29.6%. Little's missing completely at random (MCAR) test was performed to assess the missing data mechanism; this indicated that the missing data were consistent with the assumption of missing completely at random (MCAR) (chi‐square = 0.145, *p* = 0.703).

Given that proportion of missing data exceeded the prespecified 20% dropout rate, multiple imputation was conducted as a sensitivity analysis. Missing outcome data were imputed using the fully conditional specification (FCS) method, with 20 imputations generated. For binary outcomes (eradication status), logistic regression models within the FCS framework were applied. Estimates from each imputed dataset were pooled according to Rubin's rules to obtain overall effect estimates with 95% *CI*s. The pooled logistic regression analysis showed that the eradication rate was similar in the SGT group compared with the ET group [*OR* = 0.659, 95% *CI* (0.364–1.194), *p* = 0.169].

To further assess robustness, additional sensitivity analyses were performed:(i) In the complete‐case analysis, eradication rates did not differ significantly between groups [*OR* = 0.620, 95% *CI* (0.328–1.173), *p* = 0.139]. Logistic regression analysis adjusting for age and sex yielded similar results [*OR* = 0.640, 95% *CI* (0.337–1.223), *p* = 0.178].

(ii) In extreme‐case scenario analyses, assuming all missing participants in the SGT group were treatment failures and those in the ET group were successes, the difference remained non‐significant (*p* = 0.135). Conversely, assuming all missing SGT participants were successes and ET participants were failures also yielded non‐significant results (*p* = 0.499).

Collectively, these findings indicate that the primary results were robust across multiple assumptions regarding missing data.

### 3.5 Eradication Rates by Resistance Profiles in SGT Group

Among clarithromycin‐sensitive participants, clarithromycin triple therapy achieved a higher eradication rate (ITT 62.7%, PP 90.4%). In clarithromycin‐resistant participants, HDDT yielded a lower eradication rate (ITT 58.6%, PP 81.8%).

For rescue therapy, levofloxacin quadruple therapy achieved 80.0% (8/10) eradication in levofloxacin‐sensitive participants, while furazolidone quadruple therapy achieved 86.7% (13/15) eradication in levofloxacin‐resistant participants. Detailed outcomes by resistance profiles are shown in Table 2.

**Table 2:** *H. pylori* eradication rates according to antimicrobial susceptibility testing.

		Regimens	ITT	PP
First‐line treatment	Clarithromycin‐sensitive	Clarithromycin triple therapy	47/75 (62.7%)	47/52 (90.4%)
	Clarithromycin‐resistant	High‐dose dual therapy	99/169 (58.6%)	99/121 (81.8%)
Rescue treatment	Levofloxacin‐sensitive	Levofloxacin quadruple regimen	8/12 (66.7%)	8/10 (80%)
	Levofloxacin‐resistant	Furazolidone quadruple regimen	13/15 (86.7%)	13/15 (86.7%)

### 3.6 Compliance and Adverse Events

#### 3.6.1 Compliance

Treatment compliance (defined as medication use for ≥ 10 days) was high in both groups: 224/250 (89.6%) in the ET group and 227/244 (93.0%) in the SGT group, with no significant difference (Pearson Chi‐Square = 1.831, *p* = 0.176).

#### 3.6.2 Adverse Events

1. **First‐Line Therapy:** Adverse events occurred in 28/250 participants (11.2%) in the ET group and 21/244 participants (8.6%) in the SGT group (Pearson Chi‐Square = 0.930, *p* = 0.335). Reported events included bitter taste, taste alteration, abdominal pain, diarrhoea, constipation, nausea, insomnia, belching, rash, and headache.
2. **Rescue Therapy:** Adverse events occurred in 1/11 participants (9.1%) in the ET group and 5/25 participants (20.0%) in the SGT group (Fisher's exact test, *p* = 0.643).
3. **Total Adverse Events:** The overall incidence of adverse events was similar between the ET (11.6% [29/250]) and SGT (10.7% [26/244]) groups (Pearson Chi‐Square = 0.111, *p* = 0.739). Adverse event profiles by treatment regimens are summarised in Table 3.

**Table 3:** Adverse reactions according to different regimens.

	Rate *n* (%)	Details
Clarithromycin triple regimen (*n* = 75)	12/75 (16%)	5 bitter taste; 1 diarrhoea; 1 constipation; 2 taste alteration; 3 abdomen pain
Clarithromycin quadruple regimen (*n* = 250)	28/250 (11.2%)	1 allergic; 2 abdominal distension; 3 diarrhoea; 1 constipation; 5 nausea; 9 taste alteration; 2 abdomen pain; 1 insomnia; 1 belching; 2 bitter taste; 1 headache.
High‐dose dual therapy (*n* = 180)*	10/180 (5.6%)	2 diarrhoea; 1 nausea; 2 taste alteration; 3 abdomen pain; 1 rash; 1 allergic (in ET group for rescue treatment)
Levofloxacin quadruple regimen (*n* = 10)	2/10 (20%)	1 nausea; 1 taste alteration
Furazolidone quadruple regimen (*n* = 15)	3/15 (20%)	1 nausea; 1 dizzy; 1taste alteration

* Including 169 in the susceptibility‐guided therapy group and 11 in the empirical therapy group for rescue treatment.

## 4 Discussion

This randomised trial was conducted in a region characterised by extremely high antimicrobial resistance, with clarithromycin and levofloxacin resistance rates reaching 69.3% and 51.6%, respectively. These resistance levels significantly exceed thresholds commonly cited in international guidelines and represent a challenging microbiological environment for *H. pylori* eradication.

In this study, the PCR‐based susceptibility‐guided sequential strategy did not show superiority over empirical therapy under the prespecified assumptions; the observed between‐group difference was smaller than the prespecified 10% effect size used in the sample size calculation. The study may therefore have been underpowered to detect modest differences between strategies. Notably, sensitivity analyses under alternative assumptions for missing data yielded consistent findings, supporting the robustness of the primary results.

One of the most notable findings of this study is that empirical clarithromycin‐containing quadruple therapy achieved a high per‐protocol eradication rate despite a clarithromycin resistance rate of 69.3%. Conventionally, guidelines discourage clarithromycin use when regional resistance exceeds 15% (Malfertheiner et al. 2022; Smith et al. 2024; Chey et al. 2024). These findings indicate that resistance prevalence alone may not fully determine clinical performance of optimised multidrug regimens.

Clarithromycin resistance in *H. pylori* is primarily mediated by point mutations in the 23S rRNA gene, which reduce macrolide binding (Schulz et al. 2025). Clarithromycin‐heteroresistant *H. pylori* infection also interferes with successful therapy and can eventually lead to treatment failure (Kim et al. 2021). Amoxicillin resistance remains rare in this region, and its mechanism, targeting cell wall synthesis, is independent of macrolide resistance pathways (Yu et al. 2024; Tshibangu‐Kabamba and Yamaoka 2021). In addition, bismuth compounds exhibit direct antimicrobial activity, disrupt bacterial cell integrity, and may enhance antibiotic penetration (Reum Choe et al. 2024). These synergistic interactions likely contribute to the preserved efficacy of empirical quadruple therapy in this high‐resistance setting.

Although SGT did not improve eradication rates, PCR‐based testing remains valuable for resistance surveillance and stewardship. clarithromycin‐susceptible strains achieved excellent eradication with triple therapy (approximately 90%), thus confirming the high efficacy of clarithromycin when resistance is absent.

HDDT provided moderate efficacy (approximately 82%) in resistant cases, which was lower than previously reported in vonoprazan‐based regimens (Zhang et al. 2023). Regarding rescue therapy, levofloxacin‐based regimens showed variable efficacy even among susceptible strains, whereas furazolidone‐based quadruple therapy achieved relatively consistent results. These findings highlight the complexity of translating in vitro resistance prevalence into clinical decision‐making.

In addition to eradication efficacy, the practical advantages of PCR‐based susceptibility testing remain notable. This approach enables rapid turnaround and high accuracy, enabling same‐day therapeutic decision‐making. Furthermore, our study used a sequential design in which PCR‐based susceptibility testing guided both first‐line and rescue regimens, providing novel insights into its comprehensive clinical utility.

However, this study has certain limitations. It was conducted at a single centre, which may limit generalisability. The dropout rate was higher than expected, although sensitivity analyses produced consistent findings. The high first‐line eradication rate reduced the number of patients eligible for rescue therapy analysis, limiting power for secondary comparisons. In addition, the observed treatment difference was smaller than the prespecified effect size, thus potentially reducing power to detect modest effects.

In summary, in this randomised trial conducted in a high clarithromycin‐resistance region, susceptibility‐guided therapy did not demonstrate superiority over empirical treatment. However, the observed between‐group difference was smaller than the prespecified effect size, and the higher‐than‐anticipated dropout rate may have further reduced the effective statistical power. Therefore, the absence of a statistically significant difference should be interpreted with caution, and a modest potential benefit of susceptibility‐guided therapy cannot be definitively excluded. Notably, empirical clarithromycin‐containing quadruple therapy achieved satisfactory eradication rates despite a clarithromycin resistance rate approaching 70%, suggesting that optimised multidrug regimens may retain clinical effectiveness even in high‐resistance settings.

## Author Contributions

**Kemei Lu:** methodology, project administration, investigation, validation; writing – original draft, writing – review and editing. **Xuefei Zou:** data curation, formal analysis, visualisation, writing – review and editing. **Cuicui Lang:** methodology, resources, funding acquisition, validation, project administration, supervision, writing – review and editing. **Lina Zang:** data curation, formal analysis, visualisation, writing – review and editing. **Xuechao Yang:** investigation, writing – review and editing. **WeiWei Sang:** investigation, resources, writing – review and editing. **Jinyan Wang:** investigation; writing – review and editing. **Qian Feng:** investigation; writing – review and editing. **Ying Mu:** investigation; methodology; writing – review and editing. **Lifeng Liu:** investigation; methodology; writing – review and editing. **Chunhong Xu:** investigation; methodology; writing – review and editing. **Jingrun Zhao:** conceptualization; methodology; funding acquisition; validation; project administration; supervision; writing – original draft; writing – review and editing.

## Funding

The authors have nothing to report.

## Ethics Statement

This study was reviewed and approved by the Ethics Committee of Liaocheng People's Hospital (No. 2022190).

## Consent

Written informed consent was obtained from all participants.

## Conflicts of Interest

The authors declare no conflicts of interest.

## Supporting information

Supporting File

## Data Availability

The data that support the findings of this study are available from the corresponding author upon reasonable request. The data are not publicly available due to privacy or ethical restrictions.
