# Supplementary material for: Susceptibility‐Guided Versus Empirical Therapy for Helicobacter pylori Infection in a High Resistance Setting: A Randomised Controlled Trial
Source: Microbiologyopen. 2026 May 17;15(3):e70313. doi: 10.1002/mbo3.70313 (PMC13181152; doi:10.1002/mbo3.70313)
Supplement: Supplementary file 1 — Supporting File [file MBO3-15-e70313-s001.docx]

**Polymerase chain reaction detection of *H. pylori* infection and mutations conferring resistance to clarithromycin and levofloxacin**

Single-point mutations at positions 2142 and 2143 within the *H. pylori rrl* gene encoding the 23S ribosomal RNA component will indicate clarithromycin resistance. Mutations at positions 87, 88, 91, and 97 in the *gyrA* gene encoding the A subunit of the DNA gyrase enzyme, involved in DNA strain relief during bacterial replication, will indicate levofloxacin resistance.

According to the reference sequence of HP U27270, HP-23S forward primer (5′-ATGAATGGCGTAACGAGATG-3′) and HP-23S reverse primer (5′-ACACTCAACTTGCGATTTCC-3′) were employed to detect 23S rRNA gene mutations at positions 2142 and 2143. HP-gyrA forward primer (5′-GATCATAGGGCGCGCTTTACC-3′) and HP-gyrA reverse primer (5′- AAGTCGCCATCCCTACAGCGA-3′) were employed to detect gyrA gene mutations at positions 87, 88, 91, and 97. The PCR was performed in 25-µl reactions containing 12.5 µl of 2 × GS Taq PCR Master Mix, 2 µl template DNA, 1 µl forward primer, 1 µl reverse primer, and 8.5 µl double-distilled water (ddH2O). The PCR was carried out at 94 °C for 3 min, followed by 30 cycles of denaturing at 94 °C for 30 s, annealing at 58 °C for 30 s, extension at 72 °C for 30 s, and a final extension for 5 min at 72 °C.
